# Clinical and Laboratory Predictors of Dengue Hemorrhagic Fever in a Resource-Limited Setting: A Prospective Observational Study From Sri Lanka

**DOI:** 10.7759/cureus.97808

**Published:** 2025-11-25

**Authors:** SAM Kularatne, Kosala Weerakoon, Harshi Weerakoon, Madara M Rajapakse, Chamara Dalugama, Damsara Kularatne, Sithara Warnasooriya, Manoji Pathirage, Udaya Ralapanawa

**Affiliations:** 1 Department of Medicine, Faculty of Medicine, University of Peradeniya, Peradeniya, LKA; 2 Department of Parasitology, Faculty of Medicine and Allied Sciences, Rajarata University of Sri Lanka, Anuradhapura, LKA; 3 Department of Biochemistry, Faculty of Medicine and Allied Sciences, Rajarata University of Sri Lanka, Anuradhapura, LKA

**Keywords:** clinical predictors, dengue fever (df), dengue hemorrhagic fever (dhf), resource-limited setting, early diagnosis

## Abstract

Introduction: A prospective observational study was conducted to identify simple characteristics that can be used to predict the development of dengue hemorrhagic fever (DHF) in resource-limited settings.

Methods: All patients admitted to a tertiary care hospital in Sri Lanka with confirmed dengue infection, based on the nonstructural protein 1 antigen test during the 2017 dengue outbreak, were included. Sociodemographic, clinical, and laboratory investigation data collected during the first 10 days of fever were statistically compared between DHF and non-DHF patients using Student’s t-test, odds ratio (OR), and confidence interval (CI).

Results: Of the 490 dengue patients monitored, 244 (49.8%) developed DHF. A significantly high incidence of diabetes mellitus (OR: 2.23; 95% CI: 1.09-4.54), respiratory tract allergies (OR: 1.95; 95% CI: 1.24-3.05), recurrent upper respiratory tract infections (OR: 3.58; 95% CI: 2.29-5.59), past history of dengue (OR: 2.56; 95% CI: 1.04-6.28), urban living (OR: 1.52; 95% CI: 0.84-2.78), active lifestyle (OR: 11.44; 95% CI: 7.50-17.46), along with a rapid reduction in daily platelet (~34%) and white blood cell (WBC) (~25%) counts, and rise of serum alanine transaminase (ALT) (~67%) during the initial few days of fever were detected in DHF cases.

Conclusions: Comorbidities, living conditions, and physical activity levels appear to be associated with the development of DHF. A reduction of over 25% in platelet and WBC counts and a rise of more than 50% in ALT compared to the previous day’s values may help in the early detection of DHF.

## Introduction

Dengue, a mosquito-borne neglected tropical disease, poses serious global health, economic, and psychosocial challenges, with up to 400 million infections annually [1]. Sri Lanka has experienced high dengue endemicity with frequent major seasonal outbreaks over the past two decades, driven by shifts in circulating serotypes, with periodic massive outbreaks and high mortality occurring in three- to four-year intervals [2-5]. The World Health Organization (WHO) defines dengue fever (DF) as an acute febrile illness typically lasting two to seven days, often associated with facial flushing, skin erythema, generalized body aches, myalgia, arthralgia, and headache. The critical phase, dengue hemorrhagic fever (DHF), begins during defervescence, usually on days 3-7 of the illness. An increase in vascular permeability, along with increasing hematocrit levels, thrombocytopenia, and bleeding manifestations indicating significant plasma leakage, occurs during this phase and usually lasts 24-48 hours. Accordingly, the presence of fever or a history of acute fever, hemorrhagic tendencies, thrombocytopenia, and evidence of plasma leakage due to increased vascular permeability are the criteria used to clinically identify DHF [6]. While asymptomatic dengue infections are common, symptomatic cases typically present as either DF or DHF [7].

Predicting the progression from DF to the critical stage (DHF) is challenging and requires close monitoring and significant resources. Identifying reliable, clinically applicable predictors of DHF is essential, especially in resource-limited settings where existing predictors have limited utility [8-13]. Identifying predictors from a single dengue cohort may offer greater clinical relevance, aiding in effective management and recognition of atypical presentations.

Sri Lanka, being a country with high disease incidence, has its own national dengue management guidelines developed by the health ministry [14]. These guidelines were developed based on the WHO dengue guidelines for diagnosis, treatment, prevention, and control of DF [6]. Sri Lanka recorded the highest dengue outbreak in 2017, with over 100,000 reported cases and 400 deaths [2]. The Professorial Medical Unit of Teaching Hospital, Peradeniya (THP), in the Central Province of Sri Lanka, has been prospectively studying dengue patients for decades, and in this study, using the 2017 cohort, variations in clinicoepidemiological and laboratory parameters during the clinical course of dengue infection were evaluated to find simple and feasible predictors to identify the progression to DHF.

## Materials and methods

A prospective, hospital-based observational study was conducted in the Medical Unit of THP from May to August 2017, during the South-West monsoon season in Sri Lanka. Ethical approval was obtained from the Ethics Review Committee of the Faculty of Medicine, University of Peradeniya, Sri Lanka. All confirmed dengue patients with a positive nonstructural protein 1 (NS1) antigen test were included in the study after obtaining informed consent.

Patients presenting with clinical signs of dengue (fever, headache, body pain, and skin flush) within five days of fever onset were tested for the NS1 antigen, and some underwent additional immunoglobulin M and immunoglobulin G serology for confirmation. Other possible infections, such as leptospirosis, rickettsioses, influenza, and bacterial sepsis, were excluded clinically, along with basic investigations. Relevant laboratory investigations to exclude these conditions were performed for those who were clinically suspected (detailed clinical history and physical examination). It was customary to manage all dengue patients in the Medical Unit according to the dengue management guidelines published by the Ministry of Health, Sri Lanka [14]. According to the guidelines, if patients fell into the DF category with a platelet count of ≤130,000/mm^3^, they underwent monitoring according to the “observation chart for the management of dengue in adult patients without evidence of fluid leakage.” Accordingly, daily or twice-daily assessments of full blood counts were performed, along with six-hourly monitoring of packed cell volume (PCV) and three-hourly monitoring of blood pressure, pulse rate and pressure, capillary refill time, and respiratory rate in all patients identified as having DF. Such monitoring aimed to detect the onset of the critical phase of DHF. Additionally, in the ward, ultrasound scans were performed regularly to detect early plasma leakage. For example, abdominal ultrasounds were routinely performed one to three times daily per patient, with additional scans if warning signs appeared. Early ultrasound indicators, namely gallbladder edema and pericholecystic fluid accumulation [14,15], indicated entry into DHF. Management intensity was increased according to dengue guidelines [14] once the patient entered the critical phase, which usually lasts for 48 hours from the onset of fluid leakage [14]. Once the critical phase was detected, monitoring was intensified and recorded in the “observation chart for the management of dengue in adult patients with fluid leakage.” Hourly monitoring of fluid intake (both oral and intravenous), urine output (mL/kg/hour), blood pressure, pulse rate and pressure, capillary refill time, respiratory rate, temperature, and platelet and white blood cell (WBC) counts, along with three-hourly monitoring of hematocrit, was performed in all patients identified as being in the critical phase.

For this study, all these details were documented in a structured interviewer-administered questionnaire and transferred to an MS Excel worksheet (Microsoft Corporation, Redmond, WA) for analysis. After recovery, patients were discharged, and their records were archived for future reference.

In the data analysis, patients who recovered without entering the critical phase were classified as having DF, whereas those who developed plasma leakage before convalescence were categorized as having DHF. The full clinical course was defined as the period from the onset of fever to recovery. Statistical comparisons between the DF and DHF groups were performed using Student’s t-test for continuous data and odds ratios (ORs) with confidence intervals (CIs) for categorical data. A p value of <0.05 was considered statistically significant.

## Results

The cohort included 490 confirmed dengue cases, evenly split between DF (n = 246; 50.2%) and DHF (n = 244; 49.8%). Both groups showed similar mean ages (DF: 32.1 ± 13.5 years; DHF: 34.3 ± 14.5 years) with no significant difference (p = 0.387, Student’s t-test) and a male predominance (DF: 64.2%; DHF: 67.6%). Most dengue patients (n = 402; 82%) lived in rural or semiurban areas. The observed average duration from the onset of illness to hospital admission was three days, with a variability of about two days above or below that. According to the analysis of associated factors, type 2 diabetes mellitus (OR: 2.23; 95% CI: 1.09-4.54), respiratory allergies (OR: 1.95; 95% CI: 1.24-3.05), recurrent upper respiratory infections (OR: 3.58; 95% CI: 2.29-5.58), and prior dengue history (OR: 2.56; 95% CI: 0.04-6.28) were significantly associated with DHF. Further, DHF was significantly high among those with active lifestyles (OR: 11.44; 95% CI: 7.50-17.46). On the other hand, compared to DHF, DF was more common in rural areas (OR: 0.63; 95% CI: 0.44-0.90) and people with a sedentary lifestyle (OR: 0.09; 95% CI: 0.06-0.14). Income, blood group, and body mass index showed no significant association with disease severity (Table 1).

**Table 1:** Association between clinicoepidemiological factors and the development of DF or DHF Statistical significance p < 0.05 BMI: body mass index; CI: confidence interval; DF: dengue fever; DHF: dengue hemorrhagic fever; OR: odds ratio

Parameter	DF (n = 246)	DHF (n = 244)	OR	95% CI	p value
Social status, n (%)					
Urban	20 (8.66)	29 (13.06)	1.52	0.84-2.78	0.1682
Semiurban	71 (30.74)	82 (36.94)	1.25	0.85-1.83	0.2575
Rural	140 (60.61)	111 (50)	0.63	0.44-0.90	0.0116
Income level, n (%)					
Low	13 (6.22)	14 (6.14)	1.09	0.50-2.38	0.8261
Low-middle	115 (55.02)	124 (54.39)	1.18	0.83-1.68	0.3674
Middle	77 (36.84)	83 (36.4)	1.13	0.78-1.65	0.5217
Upper	4 (1.92)	7 (3.07)	1.79	0.52-6.18	0.3594
Lifestyle, n (%)					
Sedentary	184 (77.97)	52 (22.03)	0.09	0.06-0.14	<0.0001
Active	52 (22.03)	184 (77.97)	11.44	7.50-17.46	<0.0001
Health conditions, n (%)					
Diabetes mellitus	12 (4.88)	25 (10.25)	2.23	1.09-4.54	0.0277
Hypertension	17 (6.91)	10 (4.10)	0.58	0.26-1.28	0.1772
Bronchial asthma	16 (6.50)	11 (4.51)	0.68	0.31-1.50	0.3356
Other chronic illnesses	1 (0.41)	28 (11.48)	31.76	4.29-235.39	0.0007
Allergic reactions and infections, n (%)					
Respiratory tract allergies	38 (15.04)	64 (26.23)	1.95	1.24-3.05	0.0036
Skin and gut allergies	11 (4.47)	16 (6.56)	1.5	0.68-3.30	0.3145
Food and drug allergies	34 (13.82)	32 (13.11)	0.94	0.56-1.58	0.8189
Stings and direct allergies	27 (10.98)	38 (15.57)	1.5	0.88-2.54	0.1353
Recurrent upper respiratory tract infections	34 (13.82)	89 (36.48)	3.58	2.29-5.59	<0.0001
Past history of dengue	7 (2.85)	17 (6.97)	2.56	1.04-6.28	0.0406
Blood group, n (%)					
A	28 (21.54)	27 (1)	0.97	0.55-1.70	0.9116
B	46 (35.38)	39 (26.90)	0.83	0.52-1.32	0.4277
AB	4 (3.08)	9 (6.21)	2.32	0.70-7.63	0.1669
O	52 (40.00)	70 (48.27)	1.5	0.99-2.27	0.054
BMI, n (%)					
Underweight (<18.5 kg/m^2^)	35 (17.68)	47 (21.46)	1.44	0.89-2.32	0.1368
Normal (18.5-22.9 kg/m^2^)	68 (34.34)	82 (37.44)	1.33	0.09-1.95	0.1526
Overweight (23-27.5 kg/m^2^)	60 (30.30)	56 (25.57)	1.06	0.70-1.61	0.784
Obese (>27.5 kg/m^2^)	35 (17.68)	34 (15.53)	0.98	0.59-1.62	0.93

**Figure 1:**
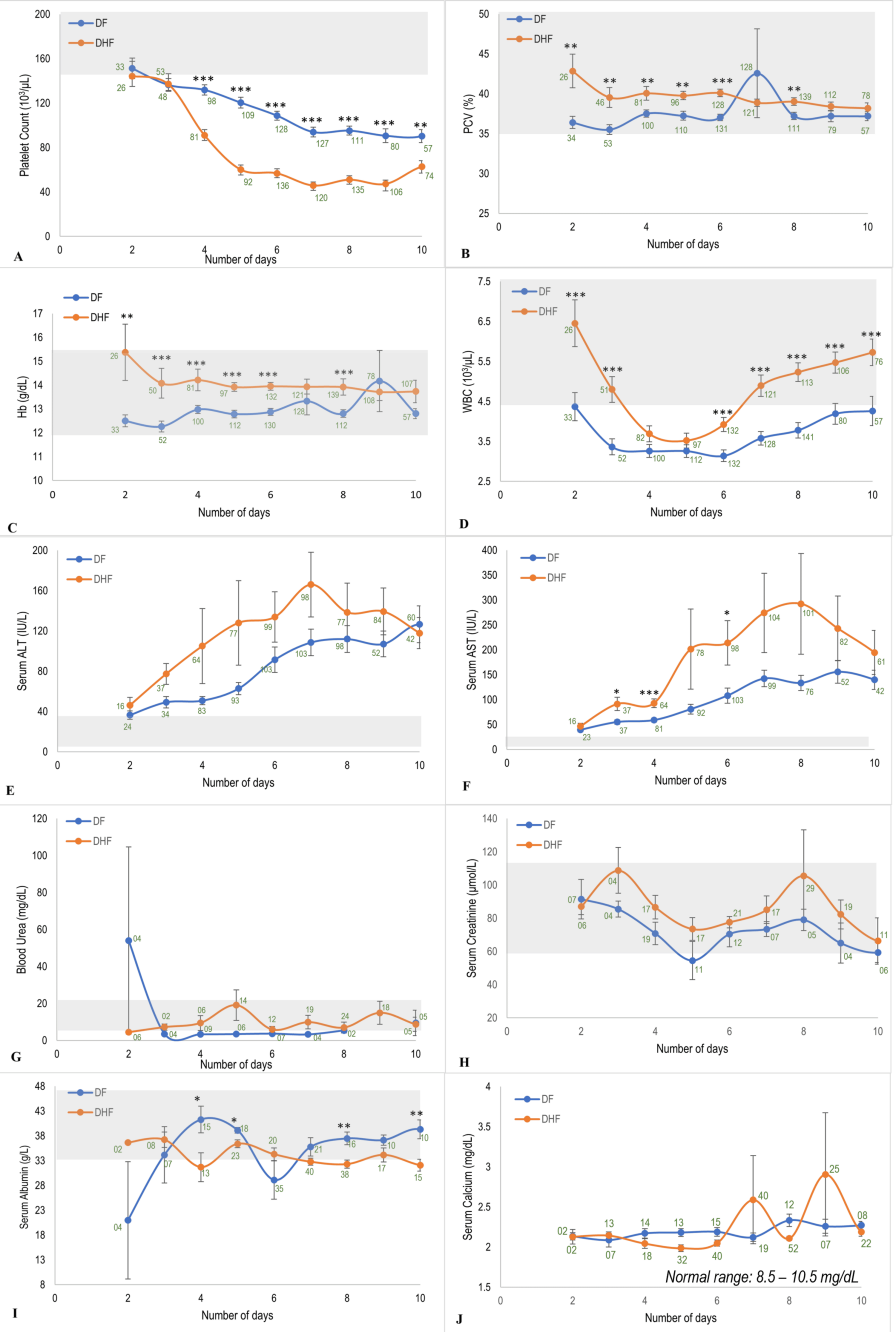
Dynamic variation of the biochemical parameters (mean ± SEM) in patients with DF and DHF over 10 days from the onset of fever. (A) Platelet count. (B) PCV. (C) Hb. (D) WBC. (E) Serum ALT. (F) Serum AST. (G) Blood urea. (H) Serum creatinine. (I) Serum albumin. (J) Serum calcium The number of patients considered in the analysis is given with each time point. The shaded area of each graph represents the normal range of the given parameter ^*^p < 0.05, ^**^p < 0.01, ^***^p < 0.001 (Student’s t-test) ALT: alanine aminotransferase; AST: aspartate aminotransferase; DF: dengue fever; DHF: dengue hemorrhagic fever; Hb: hemoglobin; PCV: packed cell volume; WBC: white blood cell; SEM: standard error of the mean

**Figure 2:**
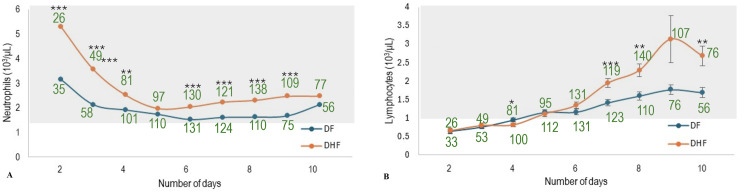
Dynamic variation of (A) neutrophils and (B) lymphocytes (mean ± SEM) in patients with DF and DHF over 10 days from the onset of fever The number of patients considered in the analysis is given with each time point. The shaded area of each graph represents the normal range of the given parameter ^*^p < 0.05, ^**^p < 0.01, ^***^p < 0.001 (Student’s t-test) DF: dengue fever; DHF: dengue hemorrhagic fever; SEM: standard error of the mean

Routine simple biochemical tests comparing DF and DHF patients over 10 days (Figure 1) showed abnormalities in mean platelet count, WBC count, serum alanine transaminase (ALT), aspartate aminotransferase (AST), and calcium levels. Approximately half of the patients (32 out of 59; 54%) had low platelet counts from day 2. DHF patients experienced a sharp decline in platelet count from the third to the sixth day of fever (Figure 1). Mean platelet counts dropped by approximately 34% each day from day 3 to 5 (137 × 10^3^/μL, 91 × 10^3^/μL, and 60 × 10^3^/μL), with significant differences (p < 0.05, Student’s t-test) between DF and DHF from day 4 onward. WBC counts remained low in the DF group throughout, whereas in DHF patients, WBC was higher, except between days 4 and 6, when no significant difference was seen. In the DHF group, WBC counts dropped rapidly by approximately 26% from day 2 (6.5 × 10^3^/μL) to day 3 (4.8 × 10^3^/μL) and 23% from day 3 to day 4 (3.7 x 10^3^/μL), followed by an increase from day 6 onward in both DF and DHF groups, with a more pronounced rise in DHF patients (Figure 1). Compared to lymphocytes, the increase in neutrophils was more pronounced in DHF than in DF. Except on days 5 and 10, the neutrophil counts were significantly high in DHF patients on all other days. On the other hand, except on day 4, no significant difference in lymphocyte count was observed between the two groups up to day 6. However, on most days, it was significantly higher in DHF patients (Figure 2), indicating differences in the dynamic changes of neutrophils and lymphocytes between DF and DHF.

In the DHF group, mean ALT rose substantially between day 2 and 5 (46-128 IU/L), though it was not significantly different from DF. AST levels were significantly higher on days 3, 4, and 6. Both enzymes showed high individual variation. Serum calcium remained low in both groups without a significant difference. PCV and hemoglobin were significantly higher in DHF patients on multiple days, whereas blood urea and creatinine showed no significant differences. Serum albumin was significantly higher in DF patients on several days. However, all these parameters fluctuated within their normal ranges.

## Discussion

This study compared basic clinical, epidemiological, and routine laboratory data between DF and DHF patients over 10 days to identify practical predictors of DHF. Most of the patients of the current cohort were admitted on day 3 of fever. Significant predictors of DHF included diabetes, respiratory allergies, recurrent respiratory infections, past dengue history, and urban residence. Additionally, a sedentary lifestyle was more associated with DF than DHF.

Contrary to typical patterns, this study found higher dengue infection rates in rural and semiurban areas compared with urban populations, despite dengue traditionally being considered an urban disease due to the habitat of its mosquito vector, *Aedes aegypti* [16]. Several factors may explain the higher number of dengue cases in rural and semiurban areas observed at THP, including its location on the outskirts of Kandy, the main city of the district, and patient referral patterns. Many residents work in the city, increasing exposure to mosquito bites; however, analyses focused on residence rather than the workplace. Although more dengue cases were reported from rural areas, urban living was associated with a higher risk of developing DHF. Urbanization, industrialization, pollution, changes in mosquito behavior, and past infections likely contribute to this risk. Additionally, an active lifestyle was associated with DHF, possibly reflecting greater exposure and reinfection among the working population, whereas a sedentary lifestyle was associated with DF.

In this cohort, diabetes, respiratory allergies, recurrent respiratory infections, and past dengue infection were significantly associated with DHF, suggesting that both metabolic and immunological factors may influence disease severity. Individuals with allergies often exhibit elevated immunoglobulin E (IgE) levels and mast cell activation, leading to increased vascular permeability [17], a feature also central to DHF [18,19]. Inflammatory mediators released by mast cells likely play a key role in the pathophysiology of DHF [20]. For example, a study in Vietnam showed that mast cell activation and their secretion of vascular endothelial growth factor and proteases contribute to the development of DHF [21]. Thus, recognizing allergic symptoms, such as pruritus, asthma, atopy, and IgE positivity, may help physicians predict the development of severe dengue and enhance patient management through risk stratification, closer monitoring, supportive care, and timely interventions.

Recurrent chest infections were significantly linked to DHF, a finding that is not discussed in the literature. This indicated a possible role of impaired or dysregulated immune responses in severe disease progression. Repeated infections may alter immune homeostasis [22], leading to dysfunctional antibody or T-cell responses that enhance susceptibility to dengue-related vascular complications [23]. Heterotypic antibodies, such as neutralizing antibodies specific to the dengue serotype and IgE in recurrent chest infections in certain immunodeficiencies [23], may trigger an exaggerated immune response, causing vascular leakage in DHF [24]. None of the patients received the dengue vaccine, as there is no approved dengue vaccine in our national immunization program. However, the ongoing dengue vaccine trials aim to induce balanced and protective immunity against all four serotypes while minimizing the risk of antibody-dependent immune enhancement, which contributes to DHF. Successful vaccination strategies could therefore reduce the incidence of severe disease by modulating exaggerated immune responses.

Among the monitored biochemical parameters, deviations in mean platelet count, WBC count, ALT, AST, and serum calcium were frequently observed. These findings align with previous studies supporting the use of hematological and serological markers to distinguish dengue from nondengue cases [25-27]. Thrombocytopenia and leukopenia are hallmarks of dengue infection [28,29]. Thrombocytopenia is known to be more severe in DHF than in DF [27]. While leukopenia is commonly associated with DF, previous studies, including the current one, have observed relatively higher WBC counts in patients with DHF compared with those with DF [27,29]. Time series analysis revealed an approximately 34% drop in mean platelet count from day 3 to 5 and a 23%-26% drop in WBC count from day 2 to 4 in DHF patients. A notable rise in serum ALT (22%-68%) was also observed from day 2 to 5. High interpatient variability in ALT and AST may reflect variability in underlying health and liver status. These findings support the importance of serial monitoring of platelet count, WBC count, and liver enzymes in the routine management of suspected or confirmed dengue cases.

The identification of predictors using a single dengue cohort further enhanced the clinical relevance of our study by ensuring consistency in case definitions, methodological approaches, data collection, and patient management. This uniformity facilitated the identification of robust associations that accurately reflect disease behavior within the studied population. Furthermore, predictors derived from a well-characterized cohort can be more readily applicable to clinical practice in similar settings and provide a reliable foundation for subsequent external validation in diverse populations. Further, this study was designed as an exploratory, hypothesis-generating investigation and was intended to screen for potential associations to guide future research, rather than to produce adjusted effect estimates. As univariate analysis is appropriate for identifying candidate predictors for subsequent confirmatory studies, the associations were reported to reflect crude relationships that do not account for confounding. Thus, these results should not be interpreted as demonstrating independent causal effects. However, this study has several limitations that need to be considered. Although the study evaluated 490 laboratory-confirmed DF patients, with 244 of them having DHF, data from all 10 days were not collected from all the patients, as some recovered and were discharged earlier to this time point. Further, the results were generated from the data obtained from the patients admitted to a single healthcare center. Relying on patient history rather than laboratory investigations to identify previous dengue infections can be considered another limitation. However, the results will be useful for identifying candidate risk factors to plan confirmatory multivariable analyses in larger, prospective cohorts to establish independent predictors of DHF. Therefore, conducting research across multiple centers with complete follow-up of all patients throughout the full course of illness is essential for further exploration of simple predictive factors for identifying DHF.

## Conclusions

The time series analysis emphasizes the clinical value of tracking trends, not just the absolute value of platelet count, WBC count, and serum ALT levels during early dengue infection. A rapid drop in platelet and WBC counts and a sharp rise in ALT early in the illness may signal progression to DHF. Notably, a rise in WBC count from day 6 may serve as an early marker of recovery, preceding platelet recovery. Thus, this study highlights that several clinicoepidemiological and basic laboratory parameters can help predict the development of DHF. Notably, the daily percentage drop in platelet and WBC counts and the percentage rise in serum ALT levels emerged as simple, feasible indicators and can be included in routine patient monitoring. These markers are routinely monitored and can be applied even in resource-limited healthcare settings after being tested through a well-designed multicenter study for early identification of high-risk patients. This can allow proactive, precision-based dengue care, timely hospitalization, close monitoring, and rational fluid management to prevent progression to DHF, dengue shock syndrome, and reduce mortality.
